# How do multi-stage, multi-arm trials compare to the traditional two-arm parallel group design – a reanalysis of 4 trials

**DOI:** 10.1186/1745-6215-10-21

**Published:** 2009-04-17

**Authors:** FM-S Barthel, MKB Parmar, P Royston

**Affiliations:** 1 Department of Biostatistics, Institute of Psychiatry, King's College London, London, UK; 2 Oncology R&D, GlaxoSmithKline, London, UK; 3 MRC Clinical Trials Unit, London, UK

## Abstract

**Background:**

To speed up the evaluation of new therapies, the multi-arm, multi-stage trial design was suggested previously by the authors.

**Methods:**

In this paper, we evaluate the performance of the two-stage, multi-arm design using four cancer trials conducted at the MRC CTU. The performance of the design at fictitious interim analyses is assessed using a conditional bootstrap approach.

**Results:**

Two main aims are addressed: the error rate of correctly carrying on/stopping the trial at an interim analysis as well as quantifying the gains in terms of resources by employing this design. Furthermore, we make suggestions for the best timing of this interim analysis.

**Conclusion:**

Multi-arm, multi-stage trials are an effective way of speeding up the therapy evaluation process. The design performs well in terms of the type I and II error rates.

## Background

Multi-arm, multi-stage (MAMS) trials employing an intermediate outcome in the early stages of a multi-stage trial with multiple research arms have been proposed as means of speeding up the evaluation of new therapies [1]. The design itself is based on discontinuing randomisation to 'poor' treatments at an early stage, and allowing through to the further stages only those treatments which show a predefined degree of advantage against the control treatment. In the early stages, the experimental arms are compared pairwise with the control according to an intermediate outcome measure. The advantage of using intermediate endpoints at interim analyses in general has been examined by Goldman et al. in simulation studies [2]. Treatment arms that survive this comparison then enter a further stage of patient accrual which ultimately culminates in pairwise comparisons against the control based on the primary endpoint. Thus, the trial is only stopped for lack of benefit, not evidence of a benefit as in many other designs [3,4]. A major issue for these designs is to assess their operating characteristics and in particular to preserve pairwise power at each stage and for the trial overall, whereby power in the second stage is conditional upon a treatment jumping the 'hurdle' at the first stage analysis.

The aim of this study was to determine whether trials which had been conducted as standard parallel group trials would have benefitted from being conducted as a multi-stage trial using the MAMS methodology. To achieve this, data from four different trials was used. There were two main objectives to this study: Firstly to assess whether using the MAMS methodology, trials would have been correctly carried on/stopped at interim analyses and secondly to quantify gains made in terms of use of resources by employing the MAMS methodology. Hence, we were concerned about the error rate at the first stage given a particular outcome at the final stage analysis. An error is defined in this context as either stopping early a trial where the final analysis would have shown an effect or carrying on a trial at the interim analysis when the final analysis would have shown no evidence of an effect. To accomplish this, a series of analyses of the intermediate outcome measure were mimicked for each of the trials using both the original trial data and bootstrapped samples from the data [5].

## Methods

### Trials

Four trials comparing treatments in different cancer sites were included in the analysis. Two of these had a positive outcome in favour of the new therapy, one was 'negative', showing no evidence of a difference between research and control arm, and one was a multi-arm trial with 'negative' and 'positive' results.

The first trial, RE01, showed considerable evidence of a treatment difference in terms of overall survival [6]. In this trial patients with metastatic renal cancer were randomised equally to subcutaneous alpha-interferon (experimental) or oral medroxyprogesterone acetate (control). A comparison of overall survival in the two groups showed a 28% reduction in the mortality rate in the alpha-interferon group (Hazard ratio = 0.72, 95% Confidence Interval 0.55–0.94). This trial used a triangular sequential design which allowed for early stopping as soon as results were conclusive.

Two trials in ovarian cancer were also re-analysed. In ICON3 [7], patients were randomised 1:2 to a research arm of paclitaxel plus carboplatin against a control arm of single agent carboplatin or CAP (cyclophosphamide, doxorubicin, cisplatin). The trial showed no evidence of an improvement of the experimental against the control arm (HR = 0.98, 95% Confidence interval 0.86 – 1.10). The second ovarian cancer trial, ICON4 [8] was designed to assess whether giving paclitaxel with platinum-based chemotherapy would benefit women with relapsed 'platinum-sensitive' ovarian cancer more than conventional platinum-based chemotherapy. The trial showed a marked improvement of the experimental over the control treatment (HR = 0.78, 95% Confidence interval 0.65 – 0.95).

Finally, the multi-arm trial FOCUS [9] in poor prognosis advanced colorectal cancer was also re-analysed. In this trial patients were randomised 2:1:1:1:1 to five treatment plans A, B, C, D and E. Regimen A (control) comprised single-agent fluorouracil until clinical disease progression, then single-agent irinotecan. B and C were fluorouracil then, respectively, fluorouracil/irinotecan or fluorouracil/oxaliplatin. D and E were fluorouracil/irinotecan or fluorouracil/oxaliplatin from the outset. Only the comparison of experimental arm C with control was found to be superior using the outcome measure of overall survival. All comparison results are given in table 1.

**Table 1:** Treatment arm comparisons in FOCUS

**Comparison**	**Hazard ratio**	**Confidence interval**
regimen A vs B	0.905	0.788 – 1.039

regimen A vs C	0.837	0.728 – 0.962

regimen A vs D	0.969	0.846 – 1.110

regimen A vs E	0.925	0.807 – 1.061

### Design of multi-stage re-analysis

We re-designed all four trials as though they had been run in a two-stage design. To do this, parameters given in table 2 and based on the original trial protocols were used to calculate the necessary number of control arm events *e*_*I *_for the first stage using the n-stage program for Stata [10]. This program is also available from the authors upon request. For trials ICON3, ICON4 and RE01, these calculations were based on using progression free survival (PFS) as the intermediate outcome. In FOCUS, however, it was decided that using PFS as an intermediate outcome was not appropriate since the randomisation was to a package of treatment both in the first line setting and at progression. Hence, this trial re-analysis employs overall survival as the outcome at both Stages 1 and 2. While the targetted hazard ratio for the intermediate and final outcome (for the alternative hypothesis) was specified as the same value in most trials, in ICON4, the trial protocol had specified a slightly lower hazard ratio for the final outcome. This value was also used for this analysis. The lower final stage significance level used for FOCUS reflects an adjustment that was made for multiple comparisons due to the number of arms considered in the trial.

**Table 2:** Parameter values for trial re-analysis based on trial protocols

**Parameter**	**ICON3**	**ICON4**	**RE01**	**FOCUS**
Number of arms	2	2	2	5

Targeted hazard ratio intermediate outcome	0.75	0.75	0.71	0.73

Targeted hazard ratio final outcome	0.75	0.74	0.71	0.73

Power at 1st stage	95%	95%	95%	95%

Power at 2nd stage	90%	90%	90%	90%

Overall power (given by nstage.ado program)	87.4%	87.4%	87.4%	87.4%

Significance level at 2nd stage (one-sided)	2.5%	2.5%	2.5%	0.5%

Overall significance level (for stage 1 *α *= 0.1)	1.39%	1.39%	1.39%	0.35%

Allocation ratio (control:experimental)	2:1	1:1	1:1	2:1:1:1:1

Intermediate outcome (median survival)	1.5 years	10 months	2.5 months	9 months

Final outcome (median survival)	3 years	23 months	10 months	9 months

An analysis on the intermediate endpoint was conducted at the point where the target number of events for the interim analysis had been 'accrued' in real time in the control group. Patients who had not 'entered' the trial at this time point were excluded from the analysis. At this analysis a hazard ratio for progression free survival was calculated and compared with a critical value for the hazard ratio determined at the design stage. This critical hazard ratio *d *was calculated as follows. Define  as the hazard ratio on the intermediate outcome in Stage 1 under *H*_0 _and let  denote the normal deviate at one-sided significance level *α*. Then, for a given probability *p *of being allocated to the control arm and a required number of control arm events 



The number of control arm events  targetted in this calculation remains the same under both the null and alternative hypothesis. Thus, this critical value gives the lower bound of a 1 -  % confidence interval around the hazard ratio under *H*_0 _of no effect [11] (p.79). This means that the one-sided 1 -  % confidence interval around  will just include *H*_0 _at a power of %. Hence if an observed hazard ratio is greater than this critical value, we can reliable exclude an effect larger than under the null-hypothesis at level . Please see the Additional File 1 for a worked example. Therefore, if the hazard ratio was smaller than this critical value a trial was counted as continuing to randomise further patients and if it was larger, the trial was counted as 'stopped' in the sense that no further randomisation would be conducted to that particular experimental arm. A hazard ratio at the end of the trial using overall survival was also calculated to obtain correlation values between the test statistics on the intermediate outcome and the primary outcome. All hazard ratios were calculated using the Cox regression model with treatment the only covariate.

To calculate an error percentage estimate, 5000 trial datasets were created based on each original trial by taking bootstrap samples with replacement from the trial dataset [5] (p.82). If this scheme were employed without any further adjustments, we would obtain a number of trials in which the overall result does not match the original trial result in terms of significance at the two-sided 5% level. However, the question we wanted to answer was whether, given a final result showing evidence of an effect of the experimental treatment, this trial would have been correctly continued at the interim analysis using the intermediate outcome measure. Hence it was decided that we would discard bootstrap samples in which the treatment effect was non-significant at the 5% level. The same applied to negative trials whereby we would discard if the bootstrapped treatment effect was significant at this level. Therefore, we employed a bootstrap sampling mechanism which was conditional on the result of the final analysis. On examining the mean of the resulting hazard ratios, we find that across the 5000 datasets, this is very close to the original trial result.

To obtain the graphical displays, patient accrual into the trial was mimicked as described above and in addition, a hazard ratio was calculated each time a new event was observed.

All analyses were conducted in Stata version 9.2.

## Results

### Results of re-analysis using trial data only

**Figure 1:**
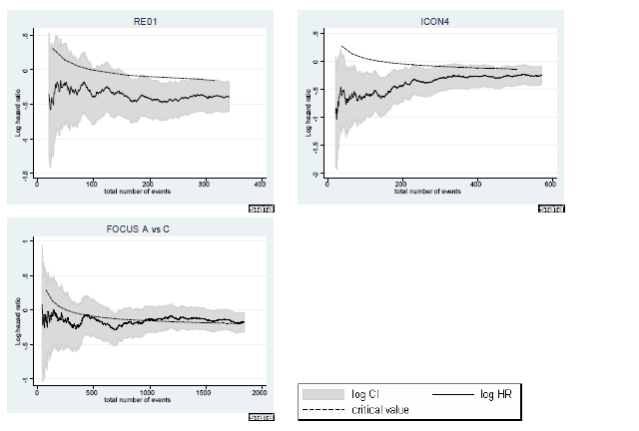
**Development of intermediate outcome hazard ratio over time in trials showing evidence of effect on primary outcome**. 95% CIs for the hazard ratio (shaded area) and the critical value for assessing whether to continue randomising further patients are given.

**Figure 2:**
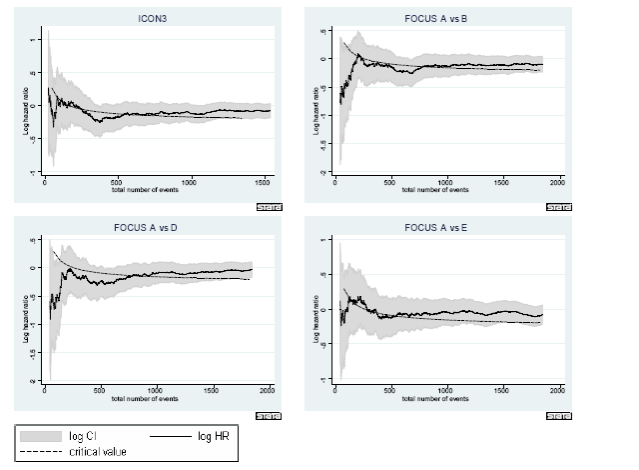
**Development of intermediate outcome hazard ratio over time in trials showing no evidence of effect on primary outcome**. 95% CIs for the hazard ratio (shaded area) and the critical value for assessing whether to continue randomising further patients are given.

In the first instance, all trials were re-analysed as described in the methods to consider what would have happened hypothetically if they had been designed as two-stage trials. The results of this analysis are displayed in tables 3 and 4 as well as Figures 1 and 2. As can be observed, for those trials/arms where the final result showed evidence of a benefit (table 3), the trial/arm would have been continued at all stages. In the trials where the final analysis showed no evidence of an effect (table 4), arms would have been stopped at some of the first stage analyses but not all. This is reflected in HR, the calculated hazard ratio at each stage which varies over the course of the trial.

**Table 3:** Re-analysis of original trial data in trials showing evidence of an effect on the final outcome.

one-sided significance	ICON4			RE01			FOCUS A vs C		
level at stage 1	*δ*_*I*_	HR	decision	*δ*_*I*_	HR	decision	*δ*_*I*_	HR	decision

0.5	1	0.553	✓	1	0.683	✓	1	0.821	✓

0.4	0.964	0.624	✓	0.964	0.729	✓	0.961	0.789	✓

0.3	0.936	0.760	✓	0.924	0.660	✓	0.930	0.921	✓

0.2	0.911	0.737	✓	0.902	0.649	✓	0.902	0.824	✓

0.1	0.885	0.760	✓	0.874	0.674	✓	0.874	0.840	✓

*δ*_*I *_gives the cut-off hazard ratio for the intermediate outcome at a given one-sided significance level, HR gives the observed hazard ratio at that point in time, ✓ denotes that the trial would have been correctly carried on to the next stage, ✗ denotes that the trial would have been incorrectly stopped at stage 1

**Table 4:** Re-analysis of original trial data in trials showing no evidence of an effect on the final outcome.

	ICON3			FOCUS						
				
one-sided significance					A vs B	A vs D	A vs E	A vs B	A vs D	A vs E
level at stage 1	*δ*_*I*_	HR	decision	*δ*_*I*_	HR			decision		

0.5	1	1.054	✓	1	0.876	0.841	1.008	✗	✗	✓

0.4	0.964	0.965	✗	0.961	0.918	0.860	0.988	✗	✗	✓

0.3	0.936	0.825	✗	0.930	0.892	0.750	0.902	✗	✗	✗

0.2	0.911	0.806	✗	0.902	0.796	0.766	0.946	✗	✗	✓

0.1	0.885	0.837	✗	0.874	0.887	0.879	0.937	✓	✓	✓

*δ*_*I *_gives the cut-off hazard ratio for the intermediate outcome at a given one-sided significance level, HR gives the observed hazard ratio at that point in time, ✓ denotes that the trial would have been correctly stopped at that stage, ✗ denotes that the trial would have been incorrectly carried on to the next stage

The figures shed further light on the developments over time. In all cases, the solid line in the middle represents the observed log hazard ratio with 95% confidence intervals given as shaded areas around it. For trials ICON4, ICON3 and RE01, log hazard ratios were calculated using the PFS outcome as this was used as the intermediate outcome in the re-design. For all arms in FOCUS, log hazard ratios were calculated based on overall survival. Each of the figures also includes the development of the log hazard ratio critical value over time, displayed as a dashed black line. The log hazard ratio in ICON4 and RE01 stays below the critical value for the entirety of the trial. However, if we consider ICON3 for example, we can identify why in 4 out of 5 analyses in the table, the trial would have been carried on despite the final result showing no evidence of a difference. The log hazard ratio is below the critical value initially, then moves above it for a short period of time after which it again moves below it. Only very much later on in the trial does it return to above the critical value again. The situation in FOCUS is similarly ambiguous in the first stages of the trial. In all comparisons the observed estimate of the hazard ratio is very close to the critical value at all times. This suggests that at best there is a very small effect of the experimental treatment over control, a long way away from the targetted hazard ratio.

### Results of re-analysis using bootstrapped data in 2 stages

Tables 5 to 8 give results for a re-analysis of all trials in 2 stages with the first two tables employing PFS as the intermediate outcome. For comparisons between the control and treatment arms for ICON3, the percentage error relates to those trials where the treatment arm would have been carried on. This is the case because the final trial result showed no evidence of a difference. This error is perhaps of not such great concern as we are continuing arms which have no clear effect at the end of trial and thus it can be argued that this is a 'conservative' error. For the comparison in ICON4 for example though, percentage error refers to the number of times when the treatment arm would have been stopped at the stage 1 analysis even though the final result was positive. This is an error of greater concern to us since we are stopping a trial which would have shown a benefit.

**Table 5:** Re-analysis over two stages of trials with final result showing evidence of a difference employing PFS as an intermediate outcome.

	ICON4			RE01		
	
one-sided sig.	e_*I*_	%	*ρ*	e_*I*_	%	*ρ*
level at stage 1		err.			err.	

0.5	75	0.04	0.27	53	3.18	0.27

0.4	97	0.20	0.29	69	4.58	0.31

0.3	125	0.03	0.36	88	1.08	0.34

0.2	162	0.26	0.39	114	0.26	0.39

0.1	221	0.12	0.44	155	0.46	0.46

e_*I *_gives the number of control arm events for the significance level at stage 1, % err. gives the percentage of trials out of 5000 which were stopped, *ρ *gives the correlation between the test statistic at stage 1 on the intermediate outcome and at the final analysis on the primary outcome

**Table 6:** Re-analysis over two stages of trials with final result showing no evidence of a difference employing PFS as an intermediate outcome.

	ICON3			
	
one-sided sig.	e_*I*_	%	*ρ*	mean time
level at stage 1		err.		saved (yrs)

0.5	116	43.40	0.27	2.33

0.4	152	46.40	0.32	2.17

0.3	194	65.64	0.35	2.02

0.2	250	87.28	0.39	1.86

0.1	339	69.02	0.47	1.53

For details see legend of table 5

**Table 7:** Re-analysis over two stages of trials with final result showing evidence of a difference using overall survival as intermediate endpoint.

	ICON4			RE01			FOCUS: A vs C		
	
one-sided sig.	e_*I*_	%	*ρ*	e_*I*_	%	*ρ*	e_*I*_	%	*ρ*
level at stage 1		err.			err.			err.	

0.5	75	0.0	0.32	53	6.32	0.44	97	9.42	0.28

0.4	97	0.0	0.46	69	3.84	0.50	125	8.4	0.30

0.3	125	0.0	0.56	88	0.62	0.57	161	32.54	0.38

0.2	162	0.0	0.70	114	0.68	0.68	206	15.44	0.42

0.1	221	0.1	0.76	155	0.04	0.88	280	16.12	0.50

For details see legend of table 5.

**Table 8:** Re-analysis over two stages of trials with final result showing no evidence of a difference using overall survival as intermediate endpoint.

	ICON3				FOCUS							
					
					A vs B			A vs D		A vs E		
one-sided sig.	e_*I*_	%	*ρ*	time	e_*I*_	%	*ρ*	%	*ρ*	%	*ρ*	mean time

level at stage 1		err.		saved (yrs)		Err.		err.		err.		saved (yrs)

0.5	116	61.44	0.31	1.89	97	68.32	0.31	82.34	0.36	43.04	0.27	1.51

0.4	152	42.44	0.36	1.67	125	58.32	0.36	81.5	0.39	50.88	0.29	1.32

0.3	194	45.4	0.41	1.48	161	56.92	0.40	90.82	0.43	57.76	0.35	1.07

0.2	250	56.46	0.47	1.25	206	77.98	0.43	88.08	0.50	34.96	0.40	0.83

0.1	339	52.56	0.54	0.83	280	35.4	0.52	44.44	0.62	16.98	0.50	0.34

For details see legend of table 5

Our analyses show that if a treatment is successful on the final outcome at the end of the trial, it has a very good chance of 'jumping' the hurdle at the intermediate stages. ICON4 and RE01 are both trials with a strong positive outcome at the end of the trial and for these two trials, the maximum error lies below 5% using PFS as the intermediate outcome and just above 6% using overall survival as the intermediate outcome. The error percentages for FOCUS comparison A vs C are greater. However, the overall trial result was not clear in either the 'positive' or 'negative' direction, which is also illustrated in figure 1. The results for ICON3 (table 6) also illustrate that since the hazard ratio is not constant over time, the trend in the error rate is also non-monotonic and counter-intuitive. In this trial, as illustrated in figure 2, a very small effect size can be observed which is close to but not at *H*_0 _and is also near the critical value. Therefore, the bootstrap gives a number of realisations which lie either side of the critical value.

Tables 7 and 8 give the results for a bootstrap analysis using the primary outcome for all stages of the trial. Interestingly, results for the error rate are not improved (and sometimes worse) when using this outcome. This suggests that the error rate is not inflated by using a different outcome at the intermediate analysis as compared to the final analysis of the trial.

The correlation displayed is the correlation between the test statistic at stage 1 and the end of the trial, i.e. the correlation between the log hazard ratio for the intermediate outcome at the time of that analysis and the log hazard ratio for overall survival at the end of the trial. In the FOCUS reanalysis, the outcome used at both stages is the final outcome. As the results illustrate, the correlation is surprisingly low for early analyses. The reason is that at a high significance level, the number of control arm events *e*_*I *_which are available for analysis is small and there is a longer time interval between that analysis and the final analysis. Furthermore, the analysis is based on a smaller subset of patients.

Tables 6 and 8 additionally give information on the mean time saved if a trial was stopped at an earlier stage for those trials with a negative overall outcome. Mean time saved was calculated by subtracting the mean time required for a stage 1 analysis in the bootstrap runs from the estimated time required for a standard parallel group trial under the design assumptions. In this case, it was assumed that had the trial only been conducted with a final analysis on overall survival, this would have been conducted at a significance level of 5% two-sided and 90% power. If an unsuccessful treatment is rejected at an early stage, savings in trial time of 1.5 – 2.3 years can be made.

Simultaneously considering the savings possible in total trial time if a trial is correctly stopped at an earlier stage and the error rate estimated for trials with a final analysis showing evidence of a difference, we can draw some conclusions on the best placing of the stage 1 analysis. This trade off between correctly and incorrectly stopping the trial early suggests that ideally, the stage 1 analysis should be placed at a significance level of 0.2 or 0.3. At this point, the error rate in both the RE01 and ICON4 re-analyses is negligible. At the same time, a saving in total trial time of on average 1 and 2 years could have been made in FOCUS and ICON3 respectively. However, more extensive studies would need to be conducted to obtain the optimal timing of the stage 1 analysis.

As the results in table 7 illustrate, FOCUS comparison A vs C would have been stopped early in some cases even though the overall trial result at the end of the trial could be viewed as being at the 'margins' of statistical significance. To circumvent this problem, it is anticipated that in a MAMS trial design, patients in those arms which were dropped at an earlier stage will still be followed up and the data analysed at a later stage. Table 9 illustrates what the result of the final analysis would have been if the FOCUS trial/arm had been stopped early and analysed at a later date. This later analysis uses mature data from the FOCUS trial in which nearly all patients had an event at the point of analysis. However, only patients accrued by the time of the relevant first stage analysis were included in the final analysis. While the results for comparisons A vs B, A vs D and A vs E are in line with the analysis given in table 1, comparison A vs C is only marginally significant if a late stage 1 analysis is conducted (HR of 0.86 if the first stage analysis is conducted at a significance level of 0.1).

**Table 9:** FOCUS results of final analysis after early stopping at stage 1.

one-sided sig. level	A vs B		A vs C		A vs D		A vs E		Power for
at recruitment stop	HR	95% CI	HR	95% CI	HR	95% CI	HR	95% CI	final analysis

0.5	0.93	(0.76–1.13)	0.88	(0.72–1.08)	0.96	(0.78–1.17)	1.03	(0.85–1.26)	90%

0.4	0.94	(0.78–1.14)	0.87	(0.72–1.05)	0.97	(0.81–1.18)	1.04	(0.86–1.25)	> 90%

0.3	0.89	(0.74–1.06)	0.88	(0.73–1.05)	0.94	(0.79–1.13)	0.97	(0.81–1.16)	> 90%

0.2	0.92	(0.77–1.09)	0.93	(0.78–1.10)	0.99	(0.84–1.17)	1.03	(0.87–1.22)	> 90%

0.1	0.89	(0.76–1.04)	0.86	(0.73–1.00)	0.99	(0.85–1.15)	0.97	(0.83–1.13)	> 90%

n/a – actual analysis	0.91	(0.81–1.06)	0.84	(0.73–0.96)	0.97	(0.85–1.11)	0.93	(0.81–1.06)	n/a

This analysis was carried out on mature data for the primary endpoint including only those patients recruited up to the time of the first stage analysis. HR – hazard ratio on primary outcome (overall survival), CI – 95% confidence interval around the hazard ratio, Power for final analysis – this was calculated for a targetted alternative of 0.73 (as specified in the protocol)

## Conclusion

Currently, according to FDA estimates [12], about 90% of agents entering Phase I do not succeed at Phase III. This is set against advances in our knowledge about pathways connected to cancer development and metastases and hence the availability of more agents for testing in clinical trials. After passing Phase I/II, the success rate of cancer Phase III trials is still only about 50%. However, if we for example employ a multi-arm, multi-stage design with four experimental regimens and one control, the probability of having at least one successful agent at the end of the trial increases to 87%. This calculation assumes independence of all experimental arms. Therefore, this type of trial design uses resources more efficiently.

In this design we propose to target the event rate in the control arm  rather than the number of events in all arms combined. There are two reasons for this approach. Firstly, an event rate different to that anticipated for the trial overall could either arise due to a different underlying event rate in all arms or due to a hazard ratio different to that targeted initially. This level of ambiguity is removed by using the control arm event rate as the deciding factor for when to conduct the analysis. Secondly, when more than one experimental arm is recruited to, it is unlikely that we shall observe the same hazard ratio in all comparisons, giving different total numbers of events for each comparison. However, the calculation for the overall number of events assumes the same event rate in all comparisons in the experimental arms.

We have demonstrated that using the MAMS designs, significant savings can be made in terms of trial time if a treatment does not prove to be effective over the course of the trial. In this case, the trial re-analyses have demonstrated that around 50% of all bootstrapped trials would have been rejected at Stage 1, regardless of when this intermediate stage was conducted. Greater savings can thus be made if a MAMS trial is designed with three or more stages as the probability of early rejection increases.

Trials with good evidence of an effect on overall survival jump all of the intermediate hurdles with very high probability. For ICON4, this was found to be as high as 100%. However, these methods do come at a cost. As the re-analysis of FOCUS comparison A/C has shown, trials or treatment arms with very small treatment effects do risk being discontinued earlier on during the trial. To alleviate this problem in employing this design at the MRC CTU we follow patients up further on discontinued arms while randomisation to these arms is stopped. Thus, these arms will also be analysed on the primary outcome at a later date albeit with reduced power in some instances, depending on the maturity of the data, so at least an estimate of the effect size can be obtained.

Our re-analyses not only demonstrate the efficiency of the MAMS design in general, but also explore the best timing of the first stage analysis. The results suggest that the first stage is ideally placed at a significance level between 0.2 and 0.3 when the trade-off between the errors for correctly and incorrectly stopping is considered. This was the point at which the error rate in the RE01 re-analysis for example became negligible, both for PFS and overall survival as the intermediate endpoint. However, this is a practical recommendation only and does not reflect an optimal design as could be obtained from a simulation study.

A further observation in this study was the behaviour of the correlation between the test statistics on the intermediate and primary outcome. If the first stage is conducted early on with a very small number of control arm events, this correlation coefficient is generally low, around 0.3. It increases over time and reaches values in the range of 0.5 to 0.7 for a very late first stage analysis.

This type of analysis would ideally be done as a simulation study, taking account of all possible trial scenarios. However, we believe that the much simpler conditional bootstrap approach that we employed is adequate. In this analysis, we needed to be able to distinguish between the error of stopping a trial early conditional on the final result showing good evidence of an effect, and the error of continuing a trial conditional on the final result showing no evidence of an effect. Hence we used a conditional bootstrap rather than the standard version since our interest was in the error conditional on a particular final outcome.

In this design no adjustment for multiple testing is made to the type I error at the interim stages. There are a number of reasons for this: i) early stopping for efficacy where the issue of type I error is perhaps more acute, is not incorporated in the design, ii) the significance level at each stage has a screening role only and is set close to 0.5 to ensure an early first stage look and iii) the overall significance level is bounded above by the significance level chosen for the final stage. If desired, this final stage significance level may be adjusted according to the number of experimental arms considered. In fact, as the methods we present are for stopping arms for lack of benefit it may be appropriate to adjust the type II error for multiple comparisons at each stage. However, in our experience this issue is typically not considered.

When analysing the trial at the intermediate stages, power may be increased by including covariates. Since the early stages will contain few patients, the trial population across the arms is more likely to be unbalanced in terms of potentially confounding covariates such as age. Including these known influential covariates in the analysis may increase the robustness of the results.

## Competing interests

FB has been employed by GlaxoSmithKline while revising the manuscript.

## Authors' contributions

FB carried out the analysis and drafted the manuscript. MP and PR were involved in discussions regarding the analysis methods and helped to draft the manuscript. All authors read and approved the final manuscript.

## Supplementary Material

Additional file 1 **Appendix: worked example**. An appendix containing a worked example for the calculation of the control arm number of events and critical hazard ratio. Click here for file
