# Supplementary material for: How do multi-stage, multi-arm trials compare to the traditional two-arm parallel group design – a reanalysis of 4 trials
Source: Trials. 2009 Apr 17;10:21. doi: 10.1186/1745-6215-10-21 (PMC2680399; doi:10.1186/1745-6215-10-21)
Supplement: Additional file 1 — Appendix: worked example. An appendix containing a worked example for the calculation of the control arm number of events and critical hazard ratio. [file 1745-6215-10-21-S1.pdf]

Appendix: worked example

In this appendix we illustrate the calculation of the control arm number of events as well as the critical hazard ratio using the ICON3 trial as an example. We refer the reader to table 2 for the trial parameters. In the calculations, a one-sided Stage 1 significance level $\alpha = 0.5$ is assumed. The size of this significance level determines the timing of the Stage 1 analysis - a higher significance level places the analysis at an earlier time point.

In a first step, the number of control arm events e_I required at each of the stages is calculated. For the first stage e_I is given by

$$\begin{aligned} e_I &= \frac{2(z_{\alpha_i} - z_{1-\beta_i})^2}{(\ln \Delta_I^1 - \ln \Delta_I^0)^2} && (1) \\ &= \frac{2(0 - 1.645)^2}{(\ln(0.75) - \ln(1))^2} && (2) \\ &= 116 && (3) \end{aligned}$$

The number of events at the final stage may be calculated in a similar manner, substituting the relevant parameters. Following this, the critical value for this stage can be calculated as follows:

$$\begin{aligned} \ln \delta_{I_1} &= \ln(\Delta_{I_1}^0) + z_{\alpha_{I_1}} \sqrt{\left(\frac{p}{e_{I_1}}\right)} \\ &= \ln(1) + 0 * \sqrt{\left(\frac{2/3}{116}\right)} \\ &= 1 \end{aligned}$$
